# Emergency pancreaticoduodenectomy for traumatic pancreaticoduodenal injury with celiac axis stenosis

**DOI:** 10.1093/jscr/rjag1022

**Published:** 2026-11-04

**Authors:** Takahiro Oishi, Takashi Muroya, Fumiko Nakamura, Kouhei Ueda, Keisuke Tamagaki, Yoshihiro Nakamura, Atsunori Onoe, Kazuhito Sakuramoto, Sohei Satoi, Yasushi Nakamori

**Affiliations:** Department of Emergency and Critical Care Medicine, Kansai Medical University Medical Center, Moriguchi 570-8507, Japan; Department of Emergency and Critical Care Medicine, Kansai Medical University Medical Center, Moriguchi 570-8507, Japan; Department of Emergency and Critical Care Medicine, Kansai Medical University, Hirakata 573-1010, Japan; Department of Emergency and Critical Care Medicine, Kansai Medical University, Hirakata 573-1010, Japan; Department of Emergency and Critical Care Medicine, Kansai Medical University Medical Center, Moriguchi 570-8507, Japan; Department of Emergency and Critical Care Medicine, Kansai Medical University, Hirakata 573-1010, Japan; Department of Emergency and Critical Care Medicine, Kansai Medical University, Hirakata 573-1010, Japan; Department of Emergency and Critical Care Medicine, Kansai Medical University, Hirakata 573-1010, Japan; Department of Pancreato-Biliary Surgery, Kansai Medical University, Hirakata 573-1010, Japan; Department of Emergency and Critical Care Medicine, Kansai Medical University, Hirakata 573-1010, Japan

**Keywords:** celiac axis stenosis, median arcuate ligament, pancreaticoduodenectomy, pancreaticoduodenal injury

## Abstract

Emergency pancreaticoduodenectomy (PD) for trauma is technically demanding, and unrecognized celiac axis stenosis (CAS) may compromise hepatic arterial inflow after gastroduodenal artery (GDA) division. A 59-year-old man presented in hemorrhagic shock after blunt abdominal trauma. Computed tomography demonstrated destructive pancreaticoduodenal injury, active bleeding, and proximal CAS with a hooked configuration suggesting median arcuate ligament (MAL) compression. After inferior pancreaticoduodenal arterial embolization, superior mesenteric arteriography showed retrograde filling of the celiac territory. Persistent hemodynamic instability necessitated emergency laparotomy and PD. Temporary GDA clamping markedly weakened common hepatic artery pulsation, confirming collateral-dependent hepatic arterial inflow. MAL release promptly restored pulsation, allowing PD with immediate reconstruction. The patient was discharged on postoperative day 34. In emergency PD for trauma, CAS or retrograde celiac filling should prompt temporary GDA clamping before irreversible division to guide MAL release or another inflow-preserving strategy.

## Introduction

High-grade pancreaticoduodenal injuries (American Association for the Surgery of Trauma Organ Injury Scale [AAST-OIS] grade IV–V) occasionally require pancreaticoduodenectomy (PD), a technically demanding procedure associated with substantial mortality in trauma patients [1–5]. Celiac axis stenosis (CAS), often related to median arcuate ligament (MAL) compression, is present in ~7% of asymptomatic individuals, and MAL compression has been reported in 2%–7.6% of patients undergoing PD [6, 7]. In CAS, hepatic arterial inflow may depend on retrograde flow from the superior mesenteric artery through the pancreaticoduodenal arcades and gastroduodenal artery (GDA). GDA division can interrupt this collateral pathway and cause ischemia of the liver and other celiac-supplied organs [7, 8]. However, recognition of collateral-dependent hepatic arterial inflow during emergency PD for trauma has rarely been described.

We report a case of traumatic pancreaticoduodenal injury requiring emergency PD, in which temporary GDA clamping demonstrated collateral-dependent hepatic arterial inflow and prompted MAL release before irreversible GDA division.

## Case report

A 59-year-old man with hypertension presented in shock after a single-vehicle truck crash with a steering wheel injury. On arrival, the patient’s vital signs were as follows: Glasgow Coma Scale score, 13; heart rate, 73 beats/min; blood pressure, 67/39 mmHg; respiratory rate, 36 breaths/min; and oxygen saturation, 100% on 10 L/min of supplemental oxygen. Focused assessment with sonography for trauma demonstrated free fluid in Morison’s pouch, and the lactate level was 5.6 mmol/L.

After intubation, contrast-enhanced computed tomography (CT) demonstrated hypoenhancement of the pancreatic head and third portion of the duodenum, a peripancreatic hematoma with active contrast extravasation, and focal proximal CAS with a hooked configuration suggesting MAL compression (Fig. 1). Because his blood pressure deteriorated, resuscitative endovascular balloon occlusion of the aorta (REBOA) was deployed, followed by emergent angiography. Active extravasation from the inferior pancreaticoduodenal artery was embolized with absorbable gelatin sponge with cessation of contrast extravasation. Superior mesenteric arteriography demonstrated retrograde opacification of the celiac territory, and celiac arteriography was technically difficult (Fig. 2). Hemodynamic instability persisted despite embolization, and laparotomy was undertaken with intermittent REBOA support.

**Figure 1:**
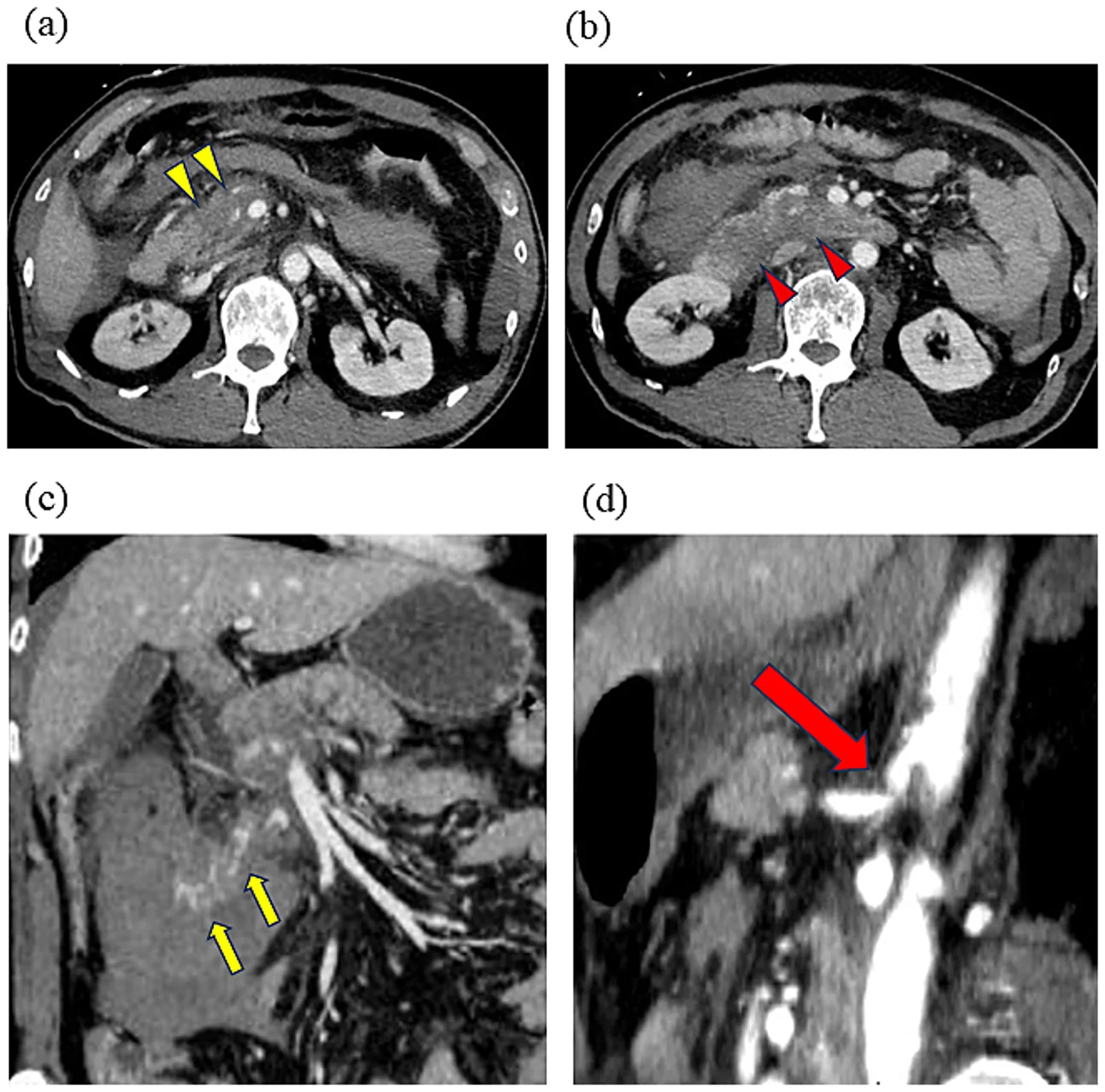
Contrast-enhanced CT on admission. (a) An axial image shows hypoenhancement of the pancreatic head (arrowheads). (b) An axial image shows hypoenhancement of the third portion of the duodenum (arrowheads). (c) A coronal reconstructed image shows a hematoma around the pancreatic head with contrast extravasation (arrows). (d) A sagittal reconstructed image shows celiac axis stenosis (arrow).

**Figure 2:**
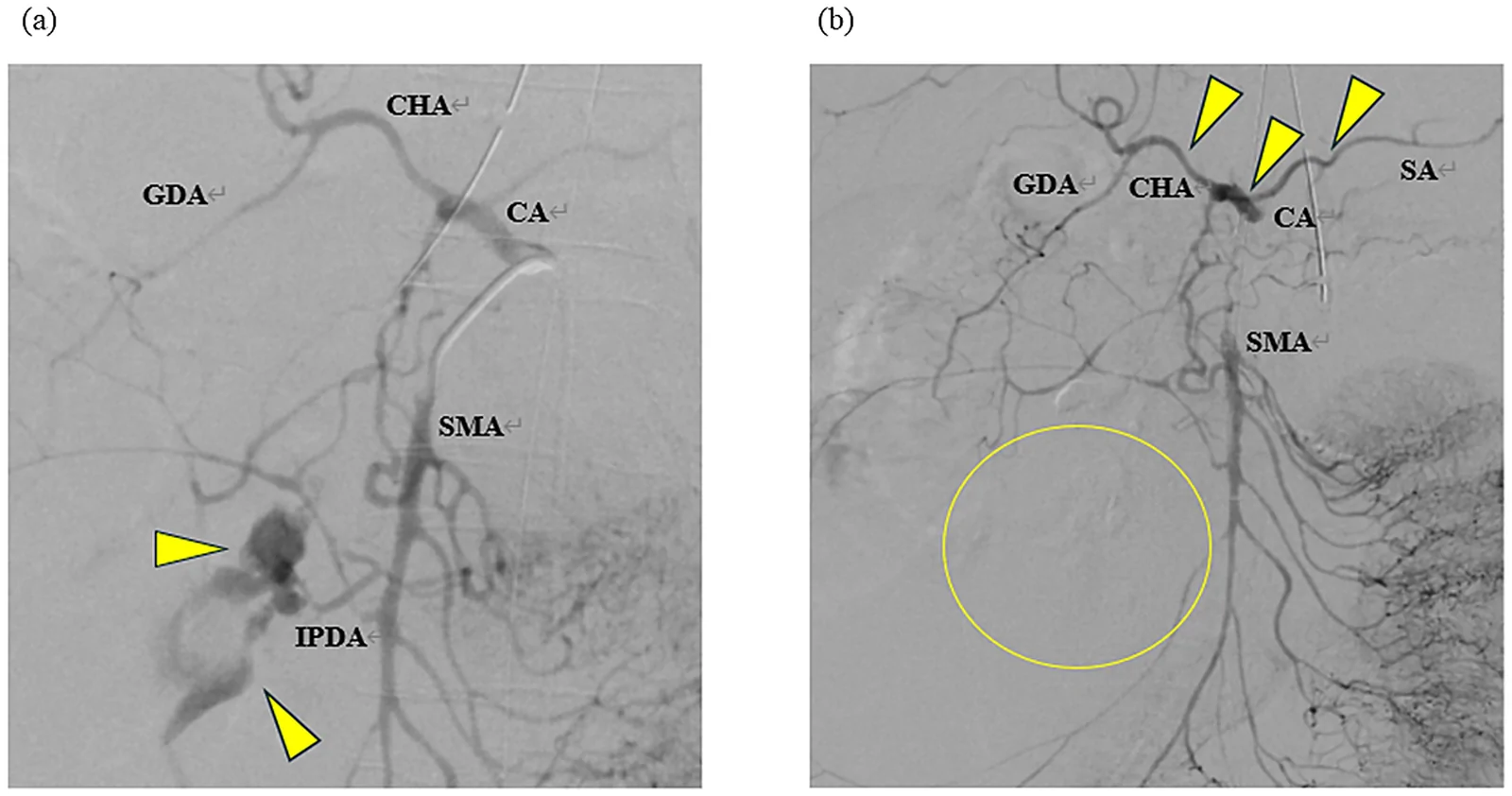
Angiography of the SMA. (a) SMA arteriography shows active contrast extravasation (arrowheads) from the inferior pancreaticoduodenal artery. (b) After embolization, the contrast extravasation disappeared (circled ). SMA arteriography also demonstrates retrograde opacification of the celiac trunk with visualization of the common hepatic, celiac, and splenic arteries (arrowheads). CA, celiac artery; CHA, common hepatic artery; GDA, gastroduodenal artery; IPDA, inferior pancreaticoduodenal artery; SA, splenic artery; SMA, superior mesenteric artery.

At the start of laparotomy, the pH was 7.15, base excess was −11.7 mEq/L, and lactate level was 8.4 mmol/L. Coagulation tests showed a prothrombin time–international normalized ratio of 1.37, fibrinogen level of 158 mg/dL, and platelet count of 48 × 10^9^/L. Laparotomy revealed massive hemoperitoneum and severe pancreaticoduodenal transection involving the uncinate process and the third portion of the duodenum, with exposure of the duodenal mucosa (Fig. 3a). The injuries were classified as AAST–OIS grade IV pancreatic and grade V duodenal injuries and were not amenable to organ-preserving repair; therefore, PD was required.

**Figure 3:**
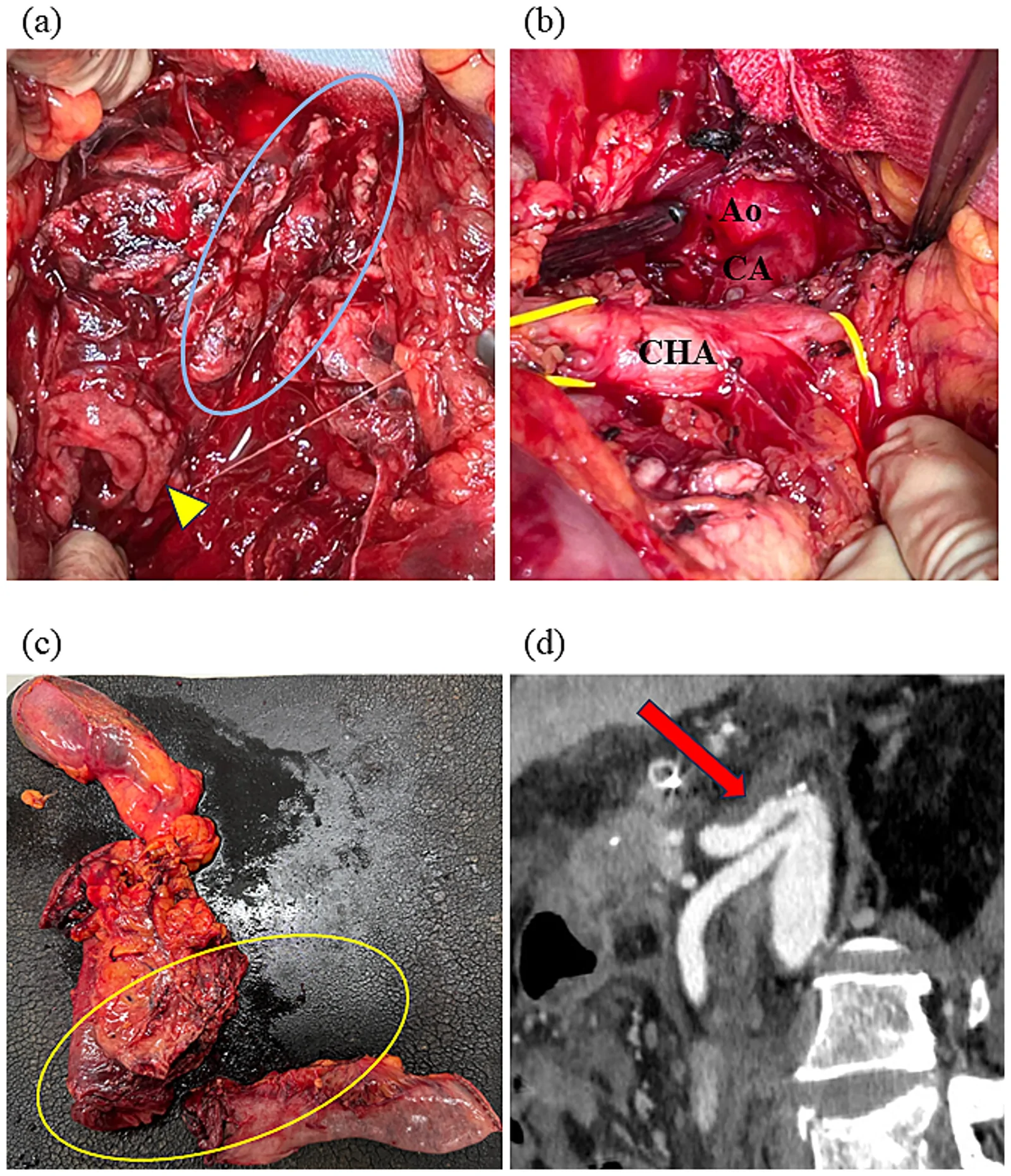
Intraoperative findings, resected specimen, and postoperative CT images. (a) Transection of the uncinate process (circled ) and the exposed duodenal mucosa (arrowhead). (b) After division of the median arcuate ligament, the celiac artery compression was relieved. (c) On the resected specimen, transection was identified at the uncinate process and at the inferior duodenal flexure (circled ). (d) Postoperative CT shows improvement of celiac artery stenosis (arrow). Ao, aorta; CA, celiac artery; CHA, common hepatic artery.

Before irreversible GDA division, temporary GDA clamping markedly weakened the palpable pulsation of the common hepatic artery (CHA), demonstrating dependence of hepatic arterial inflow on the pancreaticoduodenal collateral circulation. The clamp was released, and the MAL was exposed and divided. CHA pulsation promptly returned and remained adequate during repeat GDA clamping (Fig. 3b). PD was then completed, and the resected specimen confirmed disruption of the pancreatic uncinate process and inferior duodenal flexure (Fig. 3c). After resection, hemorrhage control was achieved, the haemodynamics stabilized, and acidosis improved (pH 7.36). Reconstruction was then performed using a modified Child procedure with modified Blumgart pancreaticojejunostomy. The operative time was 366 min with estimated blood loss of 9743 ml. Transfusions from arrival to closure totaled 24 units of red cell concentrate, 26 units of fresh frozen plasma, and 30 units of platelet concentrate. Associated injuries included an L3 compression fracture and fractures of the right first through fourth metatarsals. The Injury Severity Score was 30, the Revised Trauma Score was 6.084, and the Trauma and Injury Severity Score-predicted probability of survival was 55.5%.

The patient was extubated on postoperative day 2. Apart from delayed gastric emptying and a biochemical leak, no major postoperative complications occurred. Postoperative CT showed improvement in CAS (Fig. 3d) and he was discharged home on postoperative day 34.

## Discussion

Although MAL release after a positive GDA clamping test is an established strategy in elective PD with CAS, recognizing this hemodynamic risk during emergency trauma management is challenging because vascular assessment must be integrated with resuscitation and hemorrhage control [7, 8]. In this case, CAS on admission CT and retrograde celiac filling on superior mesenteric arteriography performed for hemorrhage control prompted assessment of hepatic arterial inflow before GDA division. Temporary GDA clamping confirmed collateral dependence and MAL release restored CHA pulsation.

In trauma patients, celiac arterial injury may manifest as dissection or occlusion [9, 10]. Because preinjury imaging was unavailable, whether CAS was pre-existing or accentuated by trauma could not be determined. However, the hooked configuration of the celiac origin and prompt restoration of CHA pulsation after MAL release favored MAL-related extrinsic compression over traumatic celiac arterial injury. Regardless of its etiology, hepatic arterial inflow should be assessed by temporary GDA clamping before irreversible division. A marked reduction in CHA pulsation on direct palpation or in Doppler-assessed flow should prompt MAL release when extrinsic compression is suspected; if inflow remains inadequate, arterial reconstruction, bypass grafting, or endovascular stenting when feasible should be considered [7, 8].

Severe pancreaticoduodenal injuries are often associated with hemodynamic instability and physiological derangement; therefore, a staged damage-control approach with delayed reconstruction is generally favored in unstable patients [1]. However, immediate reconstruction may be feasible after hemorrhage control and physiological stabilization with an experienced pancreatic surgeon [4, 5, 11]. In this case, instability persisted after embolization, but hemodynamics and acidosis improved after resection. Reconstruction was therefore a case-specific decision based on intraoperative physiological recovery and should not be generalized to all unstable patients.

In emergency PD for traumatic pancreaticoduodenal injury, CAS on available imaging or retrograde celiac filling on angiography should raise concern for collateral-dependent hepatic arterial inflow. Temporary GDA clamping before irreversible GDA division can identify this risk and guide timely MAL release or another inflow-preserving strategy.

## Data Availability

Data sharing is not applicable as no new data were created or analyzed in this study.
